# *Ocimum basilicum* (kemangi) intervention on powder and microencapsulated *Spirulina platensis* and its bioactive molecules

**DOI:** 10.12688/f1000research.52394.3

**Published:** 2022-03-14

**Authors:** Y Yuliani, Putut Har Riyadi, Eko Nurcahya Dewi, Irwandi Jaswir, Tri Winarni Agustini

**Affiliations:** 1 Master's student of Department of Aquatic Resources, Faculty of Fisheries and Marine Science, Diponegoro University, Semarang, Central Java, 50275, Indonesia; 2 Department of Fish Product Technology, Faculty of Fisheries and Marine Science, Diponegoro University, Semarang, Central Java, 50275, Indonesia; 3 International Institute for Halal Research and Training, International Islamic University Malaysia (IIUM), Selangor, 50728, Malaysia

**Keywords:** Spirulina platensis, Ocimum basilicum, synergistic, bioactive compounds

## Abstract

**Background:** *Spirulina platensis* contains several bioactive molecules such as phenol, flavonoid and phycocyanin pigments. This study unveils total phenol, flavonoid, antioxidant activity, phycocyanin content and evaluated encapsulation efficiency from *Ocimum basilicum* intervention on *S. platensis*. *O. basilicum* intervention aims to reduce unpleasant odors from *S. platensis* that will increase consumption and increase bioactive compounds.

**Methods:** The intervention was carried out by soaking a *S. platensis* control sample (SP) in *O. basilicum* with a ratio of 1:4 (w/v) and it was then dried (DSB) and microencapsulated by freeze drying methods (MSB) using a combination of maltodextrin and gelatin. Total flavonoid and phenolic analysis with curve fitting analysis used a linear regression approach. Antioxidant activity of samples was analysed with the 2,2’-azino-bis-3-3thylbenzthiazoline-6-sulphonic acid (ABTS) method. Data were analysed using ANOVA at significance level (p < 0.05) followed by Tukey test models using SPSS v.22.

**Results:** The result of this study indicated that *O. basilicum* intervention treatment (DSB) has the potential to increase bioactive compounds such as total phenol, antioxidant activity and phycocyanin, and flavonoid content. Intervention of *O. basilicum* on *S. platensis* (DSB) significantly increases total phenol by 49.5% and phycocyanin by 40.7%. This is due to the phenol and azulene compounds in *O. basilicum *which have a synergistic effect on phenol and phycocyanin in *S. platensis*. Microencapsulation using a maltodexrin and gelatin coating is effective in phycocyanin protection and antioxidant activity with an encapsulation efficiency value of 71.58% and 80.5%.

**Conclusion:** The intervention of *O. basilicum* on *S. platensis* improved the total phenol and phycocyanin content and there is potential for a pharmaceutical product for a functional food and pharmaceutical product.

## Introduction

*Spirulina platensis* is a blue-green microalga that thrives in alkaline water and it has a high potential as a source of bioactive compounds with commercial importance ^1, 2^. High value compounds with interesting functional properties such as phycobiliproteins consisting of phycocyanins and allophycocyanins, carotenoids, phenolic acids, omega-3 and omega-6 polyunsaturated fatty acids, phenol and flavonoid have been identified in *S. platensis* ^3– 5^. Phenolic compounds are a source of bioactive molecules with several beneficial health effects ^6^ due to their ability to act as antioxidants ^7^, antibacterial ^8^, and antidiabetes agents ^9^. Phycobiliproteins, carotenoids and phenol present in *S. platensis* have anti-inflamatory activities ^10^, thus making them a potential functional food product ^11^.

*Ocimum basilicum*, commonly know as sweet basil or *kemangi* in Indonesia and called *rehan* in Arabic ^12^ is a popular culinary herb. *O. basilicum* is added to a variety of foods to impart a specific aroma. *O. basilicum* contains essential oils such as chavicol, linalool and eugenol, which are widely used in the food and pharmaceuticals industries ^13^. The essential oils are able to reduce unpleasant odors and replace antioxidants ^14, 15^. Besides essential oils, basil also contains phenol and flavonoid compounds which have antioxidant properties ^16– 18^.

Microencapsulation is a technique used to coat a material to protect the material from outside factors, as well as ease handling of the material. The most important factor in encapsulation is the type of coating used. The encapsulated material is referred to as the core, intenal phase-, or filler, whereas the walls are sometimes called shells, layers, material wall, or membranes. A microcapsule can be coated by several coatings, but only one core compound can be coated ^19, 20^.

To predict the potential for bioactivity, absorption, distribution, metabolism, and excretion of a substance, our research was performed with bioinformatics and *in silico* approaches. If we do not have special apps, certain internet-based or online resources can be used. DOCK Blaster for molecular docking prediction ^21^, MDWeb and MDMoby for molecular dynamics analysis ^22^, ADMET and DrugBank for drug database creation ^23^, as well as PreADME for ADMET tools ^24^, are some of the tools available online.

Various studies have reported the presence of bioactive compounds such as phenols and flavonoid in *S. platensis* ^25^ and *O. basilicum* ^26^. The present research aims to evaluate bioactive compounds of *O. basilicum* intervention on *S. platensis*. Firstly, total phenol, flavonoid, antioxidant activity and phycocyanin contents were evaluated. Secondly, the success of encapsulation of phenol, flavonoid, antioxidant activity and phycocyanin compounds was evaluated. The addition of these compounds was expected to reduce the amount of volatiles in *S. platensis,* which cause unpleasant odors. The third was predicting absorption, distribution, metabolism, and excretion (ADME) of phenols, azulene, flavonoids, and phycocyanin.

## Methods

*S. platensis* powder was obtained from brackish water Aquaculture Fisheries (BBPBAP) Jepara (Central Java, Indonesia), *O. basilicum* was bought from a traditional market (Semarang, Central Java). The water used was multilevel distilled water, aquabidest Otsu-WI (PT. Otsuka Indonesia, Lawang, Indonesia). The reagents and chemicals used in this study were of analytical grade (CV. Chemix Pratama, Special Region of Yogyakarta, Indonesia), maltodextrin (CV. Multi Kimia Raya, Semarang, Indonesia) and gelatin (Xian, Biof Bio-Technology, Cina). This research was conducted in the food chemistry laboratory of Diponegoro University, Semarang, Central Java, Indonesia) from January 06, 2020 up to May 29, 2020. 

### Preparation of *Ocimum basilicum* leaf extract

The *O. basilicum* was extracted using distilled water (aquabidest) following modified methods reported by Handiani *et al.* ^27^. 2000 g of fresh *O. basilicum* leaves were added to 400 ml aquabidest and ground. The slurry was then filtered by using filter fabric and the extract result was approximately 1200 ml.

### Intervention of *Ocimum basilicum* on *S. platensis*

A freeze-dried sample (DSB) and microencapsulation sample by freeze drying (MSB) of *S. platensis* were soaked with *O. basilicum* extract for 10 min with ratio of 1:4, w/v. A *S. platensis* sample with no *O. basilicum* added was used as a control (SP).

### Preparation of the intervention of *Ocimum basilicum on *Spirulina platensis* by freeze drying*

*O. basilicum* and *S. platensis* were freeze-dried using a freeze dryer (Heto Powerdry LL 1500, Germany) at a temperature of -100°C for 48 hours. The *O. basilicum* extracts were applied to *S. platensis* (DSB) in the intervention study below.

### Microencapsulation of the intervention of *Ocimum basilicum on *Spirulina platensis* by freeze drying*

This Microencapsulation was performed following the methods reported by Castro-Munoz *et al.* and Dewi *et al*. ^28, 29^ Ten percent (10%) of coating materials (64 g) a consisting maltodextrin and gelatin at a ratio of 9:1, w/w were used for microencapsulated. Then, *S. platensis* were soaked with *O. basilicum* extract were added into the mixture. Homogenization was then performed with a homogenizer (15A HG-wiseTis, Germany). *S. platensis* treated with microencapsulation freeze-dried *O. basilicum* (MSB) was used in the intervention studies below.

### Determination of total phenol content

Total phenol content was measured using modified Folin-Ciocalteu methods ^30^. Samples were sonicated for 30 minutes prior to measurement. Gallic acid was used as standard and was read at λ=739 nm using a UV-Vis spectrophotometer. In the test solution, 0.5 ml of Folin-Ciocalteu reagents and 1 ml of NaCO _3_ were added to 1 ml of sample and the solution was mixed. Samples were incubated for 10 minutes at room temperature, then diluted with aquabidest to 10 ml. The measurement results were reported in milligram (mg) and were calculated as gallic acids equivalent (GAE) per gram of sample. The result of the gallic acid calibration curve obtained equation y = 1.0677 x – 0.0022 with a value R ^2^ = 0.9915.

### Determination of flavonoid content

Measurement of total flavonoid was performed using the slightly modified aluminium chloride method ^31^. Modification was through ultrasonic treatment before measurement, the sample was sonicated for 30 minutes and quercentin was used as a standard. In the test solution, 1.0 ml of sample was mixed with 0.3 ml of NaNO _2_ (5%, w/v) and the solution was left to stand 5 minutes before 0.5 ml of AlCl _3_ (2%, w/v) was added to the test solution. Samples were neutralized with 0.5 ml of 1 M NaOH solution and the samples were incubated for 10 minutes at room temperature. Absorbance was measured at λ=310 nm. The results are presented in milligrams (mg) and calculated as quercentine equivalent (QE) per gram of sample. The result of the quercetin calibration curve obtained equation y = 0.0185 x + 0.0223 with a value R ^2^ = 0.9995.

### Phycocyanin content

40 mg of sample was added into 10 ml centrifugal tube phosphate buffer (pH 7) 100 mM; the solution was sonicated for 30 minutes and stored at 4°C overnight. Samples were centrifuged to separate the blue supernatant. Next, samples were measured for absorbance at 620 nm according to the methods described by Setyoningrum & Nur ^32^. Phycocyanin content was determined using Equation 1:


$$PC\;(\%)\;=\;\frac{\text{Abs}\;\text{x}\;\text{v}}{3,39\;\text{x}\;w\text{x}w_{dry}}\;x\;100\%\;(1)$$

Where PC is phycocyanin content, Abs is absorbance at 620 nm; v is volume of solvent (ml); 3.39 is the coefficient of C-Phycocyanin at 620 nm; w is weight of sample (mg); and w _dry_ is percentage dry weight of sample.

### Determination of antioxidant activity

The antioxidant activity of the sample was measured by 2,2’-azinobis-3-ethylbenzo-thiazoline-6-sulfonic acid (ABTS) radical according to the methods of Shalaby & Shanab ^33^. ABTS was formed by reacting 7 mM ABTS aqueous solution with 2.45 mM phosphate per sulphate in the dark for 4–16 hours at room temperature. Dilute ABTS solution with ethanol absorbance of 0.700 ± 0.05 at 734 nm was used for measurement. The photometric test was carried out with 0.9 mL ABTS solution and 0.1 mL of the tested sample mixed for 45 seconds, measurements were made immediately at 734 nm after 15 minutes. Antioxidant activity was expressed as the inhibition percentage of free radicals by the sample and was determined using Equation 2:


$$\mathrm{Inhibition}\;(\text{\%})\;=\;\frac{\text{Ab}\;-\;\text{As}}{\text{Ab}}\;x\;100\;(2)$$

Where Ab is the absorbance of the control reaction and As is the absorbance in the presence of the extract sample.

### Determination of encapsulation efficiency

Encapsulation efficiency (EE) was determined following the methods described by Ong *et al.* ^34^. Encapsulation efficiency was calculated based on total coated active compounds and free active compounds. Percent encapsulation efficiency was determined using Equation 3:


$$EE\;(\%)\;=\;\frac{\text{Total}\;\text{coated}\;\text{active}\;\text{compounds}\;-\;\text{Free}\;\text{active}\;\text{compounds}}{\text{Total}\;\text{coated}\;\text{active}\;\text{compounds}}\;x\;100\;(3)$$

Where total coated active compounds is the total active compounds such as phycocyanin, phenol, flavonoid and antioxidant in the microcapsule (MSB sample). While free active compounds is the mass of active compounds such as phycocyanin, phenol, flavonoid and antioxidant in the microcapsule (powder) surface.

Free active compounds mass was calculated as follow:

- Phycocyanin (40 mg microcapsule were washed with 10 ml of buffer phosphate)
- Total phenol (1 g microcapsule were washed with 9 ml of aquabidest)
- Flavonoid (50 mg microcapsule were washed with 5 ml of methanol)
- Antioxidant activity (20 mg microcapsule were washed with 2 ml of ethanol)

The solution were filtered using Whatman paper No.42. After filtration, the free active compounds was measured according to the same methods described for active compounds such as (phycocyanin, total phenol, flavonoid and antioxidant activity) determination.

Parameter of separation of the free active compound from the encapsulation is the solubility of the active compounds when washed by strirring for one minute.

### ADME analysis

The research was performed in two phases, namely: the first stage of accessing the PubChem server ( https://pubchem.ncbi.nlm.nih.gov/) to obtain canonical SMILE information; the next step is to use swiss ADME ( http://www.swissadme.ch/) to predict absorption, distribution, metabolism, and excretion ^35^. The BOILED Egg (Brain Or IntestinaL EstimateD permeation predictive model) methods are used for the determination of the absorption of the inhibitors in the brain and gastrointestinal tract. BOILED Egg provides a threshold (TPSA ≤ 131.6 and WLOGP ≤ 5.88) and the best representation of how far molecular structure is for well- or poorly absorbed ^36^. ADME is based on the Lipinski rule of five ^37^. The Lipinski rule of five is generally employed in accessing the drug-likeness of active compounds to prioritize compounds with an increased likelihood of high oral absorption ^38^.

### Statistical analysis

Data obtained was reported as the mean of triplicates (n=3) ± standard deviation. Parametric data was analyzed using SPSS version 22.0 (IBM, Armonk, NY, USA) ^39^. Statistical analysis was preceded by a normality test with One Sample Kolmogorov-Smirnov Test and a homogeneity test with the Levenes Test at significance level (P > 0.05). Parametric tests were carried out with One Way ANOVA at significance level (P < 0.05), followed by post hoc Tukey HSD.

## Results

**Figure 1.**
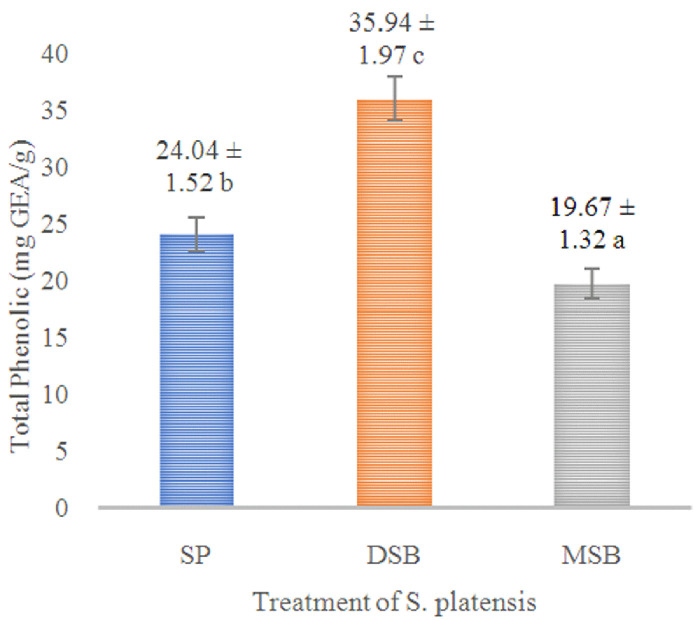
Total phenol content (n=3, mean value ± standard deviation; different superscripts indicate a significant difference). SP = *S. platensis* with no treatment, DSB = *S. platensis* treated with freeze-dried *O. basilicum*, MSB = *S. platensis* treated with microencapsulation freeze-dried *O. basilicum*.

**Figure 2.**
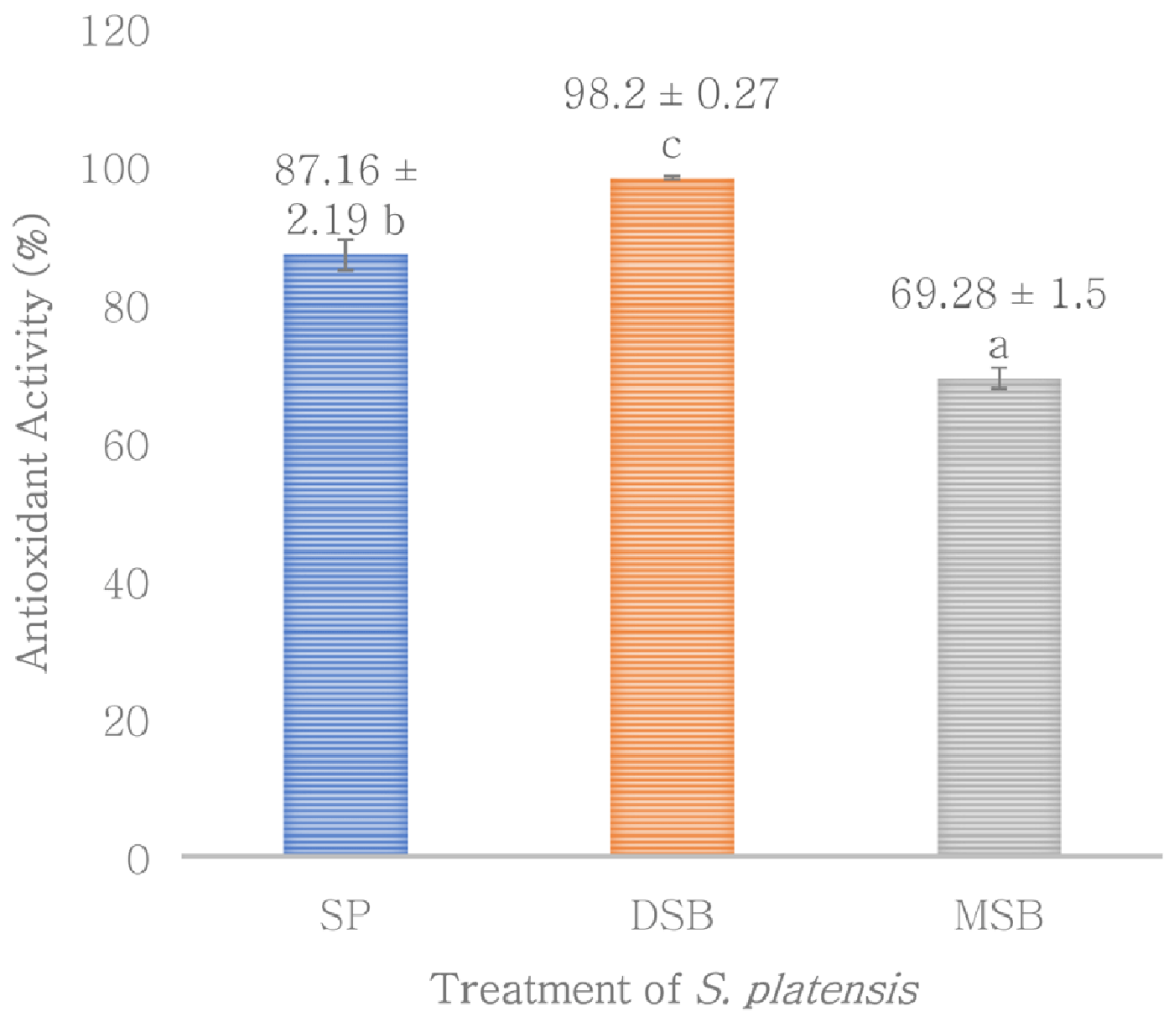
Antioxidant activity (n=3, mean value ± standard deviation; different superscripts indicate a significant difference). SP = *S. platensis* with no treatment, DSB = *S. platensis* treated with freeze-dried *O. basilicum*, MSB = *S. platensis* treated with microencapsulation freeze-dried *O. basilicum*.

**Figure 3.**
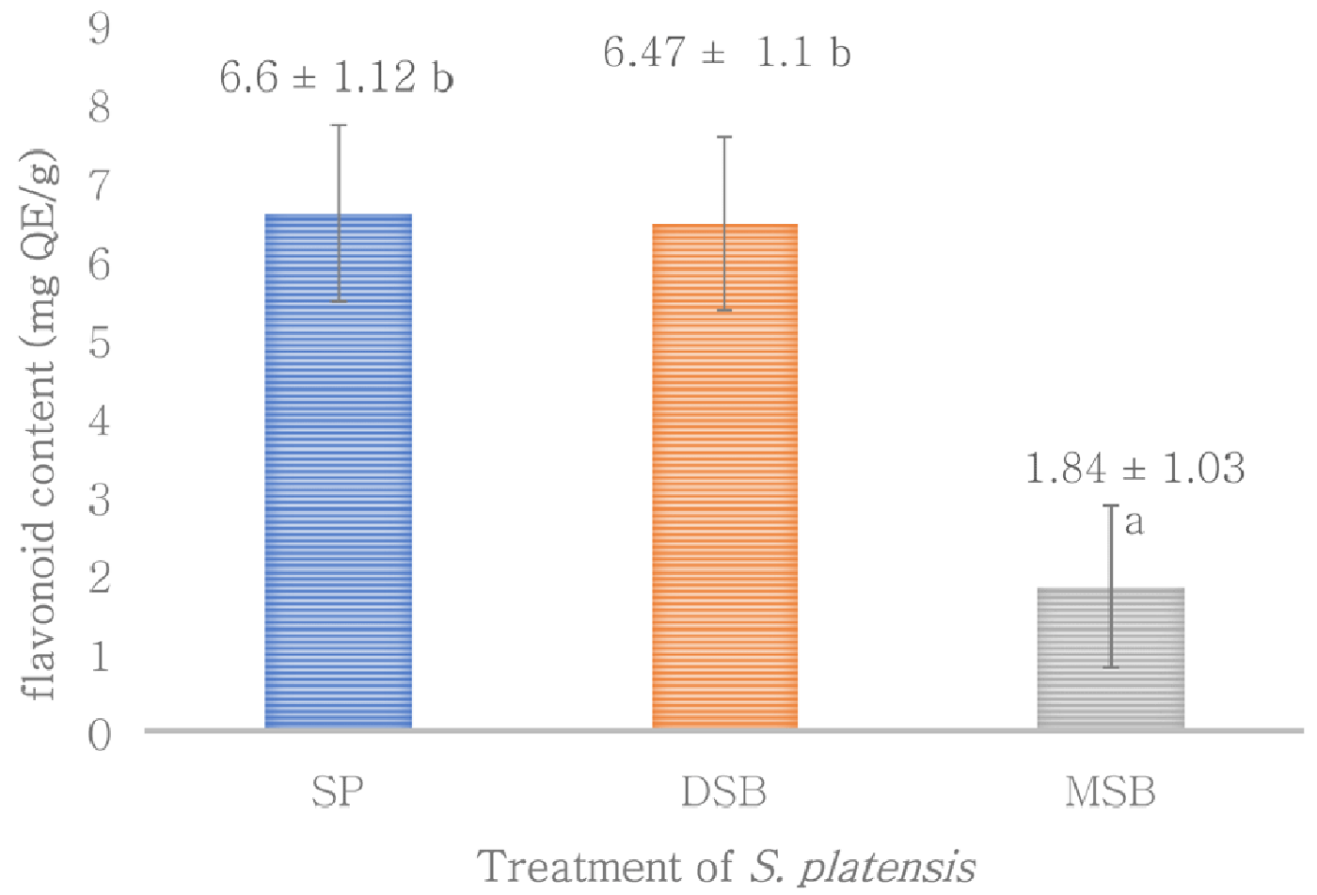
Flavonoid content (n=3, mean value ± standard deviation; different superscripts indicate a significant difference). SP = *S. platensis* with no treatment, DSB = *S. platensis* treated with freeze-dried *O. basilicum*, MSB = *S. platensis* treated with microencapsulation freeze-dried *O. basilicum*.

**Figure 4.**
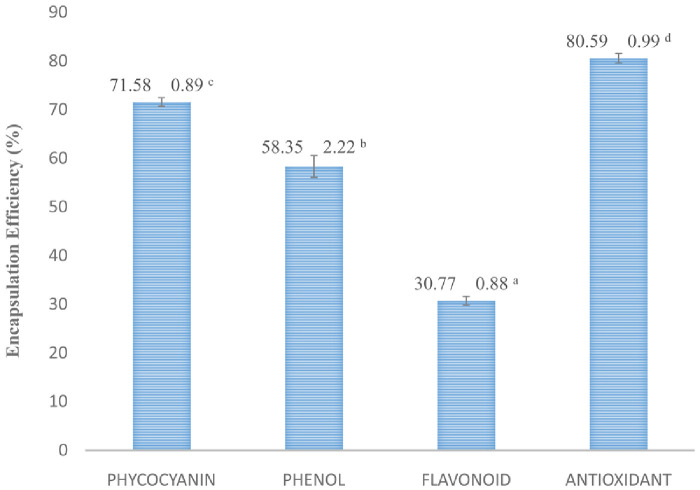
Encapsulation efficiency. (n=3, mean value ± standard deviation; different superscripts indicate a significant difference.

Total phenol, flavonoid and antioxidant activity were measured in *S. platensis* with no treatment (SP), *S. platensis* treated with freeze-dried *O. basilicum* (DSB), *S. platensis* treated with microencapsulation freeze-dried *O. basilicum (*MSB) and *O. basilicum* leaf extract (B). Phycocyanin content was measured in SP, DSB and MSB, and then encapsulation efficiency was measured on total phenol, flavonoid, antioxidant activity and phycocyanin. The DSB sample can increase the total phenol 49.50% ( Figure 1) and antioxidant activity 12.67% of *S. platensis* ( Figure 2). However, total flavonoid is not significantly different with *O. basilicum* intervention on *S. platensis* ( Figure 3). The MSB sample is an effective for protecting phycocyanin and antioxidant activity with higher value an encapsulation efficiency. However, this encapsulation is less effective of polyphenol compounds such as phenol and flavonoid as shown in ( Figure 4). *Ocimum basilicum* extract was analysed the bioactive compounds such as total phenol, flavonoid, and antioxidant activity for 117.24 ± 8.06 mg GAE/g, 7.04 ± 0.18 mg QE/g and 94.93 ± 2.24%, respectively.

**Figure 5.**
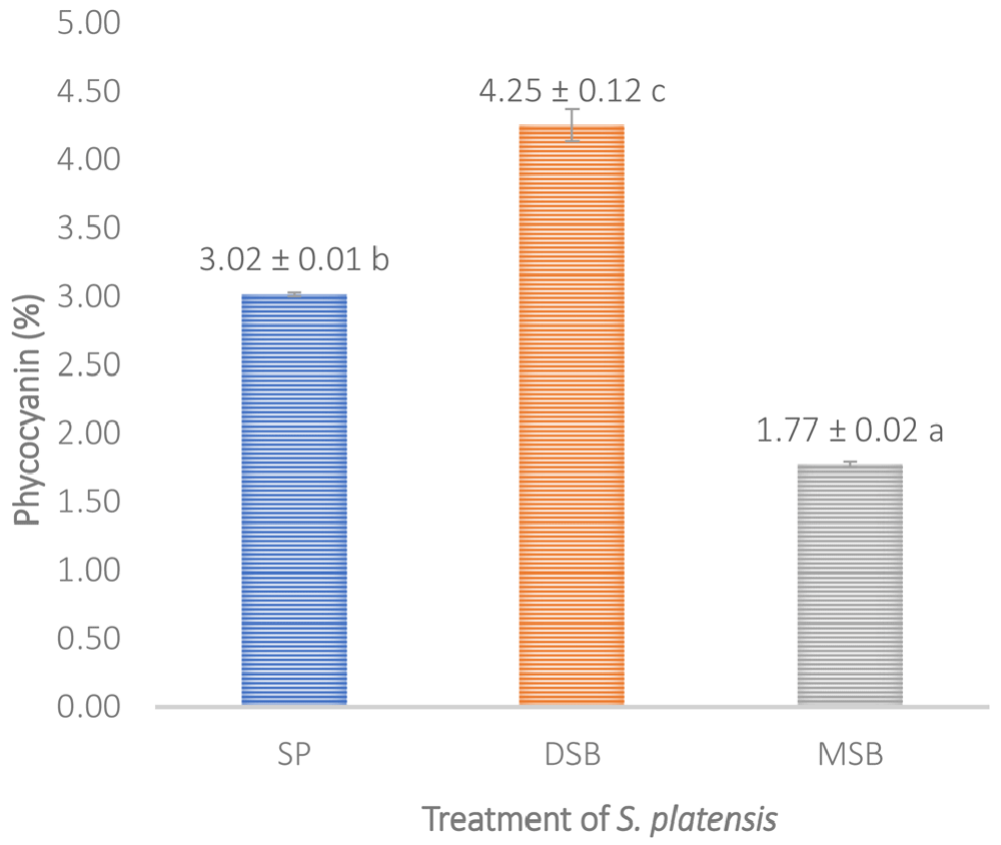
Phycocyanin content (n=3, mean value ± standard deviation; different superscripts indicate a significant difference). SP = *S. platensis* with no treatment, DSB = *S. platensis* traeted with freeze-dried *O. basilicum*, MSB = *S. platensis* treated with microencapsulation freeze-dried *O. basilicum*.

*O. basilicum* intervention can increase the levels of phycocynin in *S. platensis* 40.72% shown in ( Figure 5). *O. basilicum* intervention on *S. platensis* when extracted will make a blue ring on the surface, it is caused by compounds contained in *O. basilicum* called azulene. Raw absorbance data for bioactive compounds assays are available as *underlying data* ^40^.

## Discussion

Microalgae are a valuable source of proteins and phenol compounds. *S. platensis* is a type of microalgae with a high total phenol content ^41^. Extraction methods and the solvent used are responsible for the type and yield of phenolic compounds from algae sources ^42^. In *S. platensis*, distilled water has been reported as the best solvent for extraction of phenolic compounds with total phenol content of 43.2 ± 1 mg GEA/g ^43^. *S. platensis* powder prepared via oven drying is reported to have a broad range phenolic profile that includes gallic acid, catechin, caffeic acid, P-hydroxybenzoic acid, P-cumaric acid, ferulic acid, quercein, genistein and kaempferol ^44^. Variation in total phenol content between algae species is reportedly due to algal type, origin and growth condition of different microalgae ^45^.

Fresh *O. basilicum* leaf extract has been reported to have lower total phenol content than that which has been freeze-dried ^46^. Previous studies have reported that dried *O. basilicum* leaf extracted with methanol gave high total phenol values ^47^. The phenol compounds present in *O. basilicum* include rosmarinic, caftaric, caffeic, chicoric, p-hydroxybenzoic, p-coumaric, and protocatechuic acids. The phenol compounds in *O. basilicum* play an important role in its antioxidant activity ^48^. *S. platensis* microcapsule with the intervention of *O. basilicum* (MSB) gives low total phenol. Microencapsulation using maltodextrin and gelatin can protect polyphenol compounds ^49^.

*O. basilicum* intervention on *S. platensis* significantly increases bioactive compounds of the total phenol ( Figure 1), phycocyanin ( Figure 3) and antioxidant activity ( Figure 4), except for flavonoid content ( Figure 2). The total flavonoid content of the *S. platensis* treated with freeze-dried *O. basilicum* (DSB) was not significantly different from the control sample (SP). Previous studies have reported that total flavonoid in *S. platensis* is less than the total phenols, phenolics (1.73%) and flavonoids (0.87%) ^50^. Another study reported that the powder of *S. platensis,* which was dried in an oven at a temperature of ± 50°C, did not effect the phenolic compound quercentin, where the compound was one of the active substances of the flavonoid class ^44^. The flavonoids are considered as indispensable in a variety of medicines, nutraceutical, pharmaceutical and cosmetic applications ^51^. Flavonoids derivative compounds play an anti-inflammatory and antioxidant namely hesperidin and quercetin ^52^. The optimum for the extraction process are dry conditions compared to wet conditions. Extraction using ethanol had a higher total flavonoid content ^53^. The total flavonoid content of the *S. platensis* microencapsulated and freeze-dried tended to be low. Microencapsulation can maintain the stability of flavonoid from processing effects that cause degradation ^54, 55^.

*Spirulina platensis* could be considered as a valuable source of bioactive colored components as phycocyanin, chlorophyll, carotenoid and phenolic compounds with potent antioxidant activity ^25^. The ABTS method was chosen because it has a high level of sensitivity (99.44%) compared to the 1,1-Diphenyl-2-Picrylhydrazyl (DPPH) method (95.3%) ^33^. Total phenol and flavonoid content showed positive correlation to the antioxidant activity of *S. platensis*. Phenolic components play an important role in the antioxidant activity ^56^. Phenolic compounds are good electron donors because the hydroxyl groups can contribute to antioxidant activity ^57^. The tocopherol and phycocyanin in microalgae have potential as antioxidants in food, so that it acts as a functional food ^58^. The *S. platensis* treated with freeze-dried *O. basilicum* (DSB) showed an increase in antioxidant activity compared to *S. platensis* with no treatment (SP). Previous research explained that *O. basilicum* contains essential oils which also have potential as antioxidants ^59^. According to 60, the mixture of carotenoid pigments, chlorophyll and blue pigments such as phycocyanin of *S. platensis* produce strong antioxidants.

*Ocimum basilicum* contains 65 active compounds, and the compounds with the highest content are namely 31.6% linalool and 23.8% methylchavicol. Essential oils in *O. basilicum* have the potential as antioxidants ^13^. The essential oil of linalool significantly prevents the formation of UVB-mediated 8-deoxy guanosine, which causes oxidative damage to DNA. This is because it has the ability to prevent reactive oxygen species (ROS) and restore the balance of oxidative cells ^61^. This research indicates that there is a synergistic interaction between phycocyanin and total phenol in antioxidant activities. The high contents of total phenol ( Figure 1) and phycocyanin ( Figure 3) had a positive correlation with antioxidant activity ( Figure 4) in *S. platensis* treated with freeze-dried *O. basilicum* (DSB). *S. platensis* treated with microencapsulation freeze-dried *O. basilicum* (MSB) impart smaller values on total phenol, flavonoid, phycocyanin and antioxidant activity. This is in correlation with previous research which showed that the *S. platensis* microcapsule has antioxidant activity of 49.05% ^62^. Essential oils that play a role as an antioxidant can last for six months with a slight decrease in antioxidant activity and phenol content after microencapsulation ^63^. Treated microencapsulation can control antioxidant capacity and is a promising strategy in extending shelf life ^55^.

*S. platensis* cultivated with brackish water had a higher phycocyanin content ( Figure 3), whereas *S. platensis* cultivated in freshwater only had a 1.74% phycocyanin content ^64^. *S. platensis* cultivated with seawater has a maximum phycocyanin content ^65^. Phycocyanin is a natural blue pigment that functions as an antioxidant, anti-inflammatory and anti-carcinogenic ^66, 67^. The *S. platensis* treated with freeze-dried *O. basilicum* (DSB) impart higher levels of phycocyanin, where a combination of *S. platensis* and *O. basilicum* with a ratio of 1:5 detects the presence of azulene using gas chromatography-mass spectrometry (GC-MS) ^27^. Azulene is an aromatic compound from essential oils in *O. basilicum* ^68^, and it is a blue hydrocarbon compound that has a strong dipole moment ^27, 69^. Azulene has a small gap between the highest energy molecular orbitals (HOMO) with the lowest energy molecular orbitals that do not have electrons (LUMO) ^70^. Therefore, the presence of azulene in *S. platensis* treated freeze-dried *O. basilicum* can increase phycocyanin levels.

Previous research showed that intervention *O. basilicum* increase hedonic scale of *S. platensis*. The *O. basilicum* intervention treatment (DSB) has the best score in aroma and texture, while *S. platensis* microcapsules with the intervention of *O. basilicum* (MSB) has the best score in color and appearance ^71^. Volatile compounds that comtributed to this off-odour in *S. platensis* are geosmin, 2-Methylisoborneol and medium chain-alkanes. The intervention showed in a decrease in these volatile compounds in *S. platensis* ^72^.

Encapsulation efficiency is used to evaluate the success of a microencapsulation technique. Encapsulation using a combination of polyanion and polycation coatings such as maltodextrin and gelatin has a higher yield. This is due to the stability of the emulsion between maltodextrin and gelatin ^73^. The amount of bioactive content on the surface will reduce the value of encapsulation efficiency. This will cause the amount of bioactive compounds that are wrapped to increasingly shrink because many are attached to the surface. So that this event will damage the oxidative stability of microcapsules ^74^. The encapsulation efficiency of phycocyanin was in accordance with the results of previous studies ^75^, which is encapsulation using an alginate coating has an encapsulation efficiency value of 71.75%. The value of encapsulation efficiency in total phenols and flavonoids in *S. platensis* is effected by using liposomes or nanoliposomes in encapsulation of bioactive compounds, this is because liposome is stable at low pH and is able to withstand the time of release in the stomach, but it is less consistent in the intestine ^76, 77^. The encapsulation efficiency of antioxidant has been shown in previous research where antioxidant microencapsulation using the freeze drying method has an encapsulation efficiency value ranging from 73–86% ^78^.

Intestinal absorption and brain permeation set crucial parameters at their target site of action for any medication for its pharmacokinetics and bioavailability. Consequently, the BOILEDEgg study was used, as previously stated, to predict gastrointestinal (GI) absorption and brain access for phenol, azulene, flavonoid, and phycocyanin. The white region is the physicochemical space of the molecules most likely to be consumed by the gastrointestinal tract, whereas the yellow region (yolk) is the physicochemical space of the molecules most likely to reach the brain. The white and yolk regions are not mutually exclusive ^36^. Phenol, azulene, and phycocyanin were found to be among the well-absorbed molecules based on the study ( Table 1).

**Table 1.** Pharmacokinetics parameters of the analysis compound predicted by Swiss ADME.

Compound	GI absorption	BBB permanent	Bioavailability radar	Boiled egg
Phenol	High	Yes	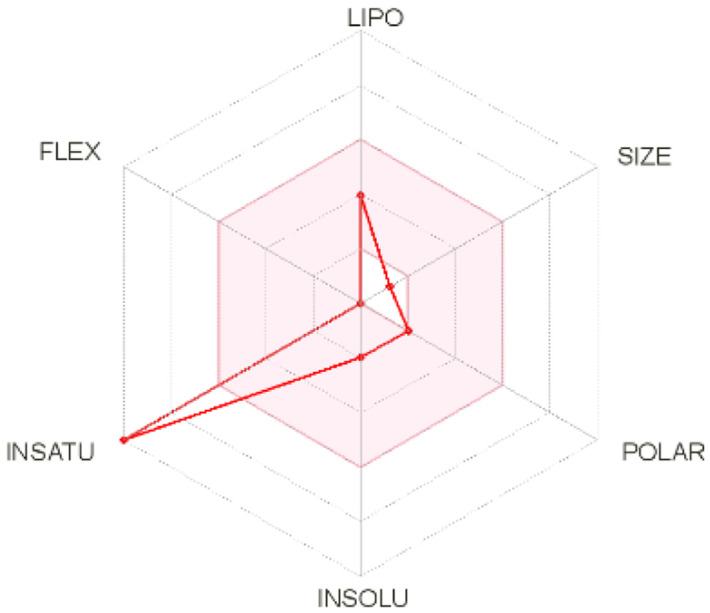	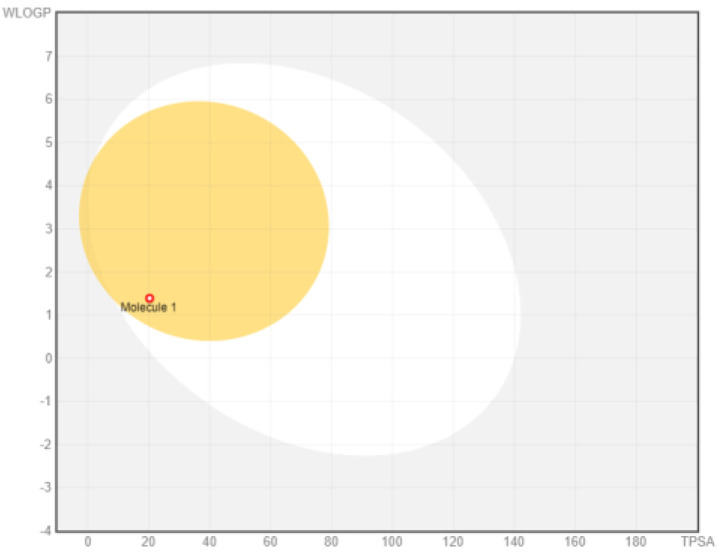
Azulene	Low	Yes	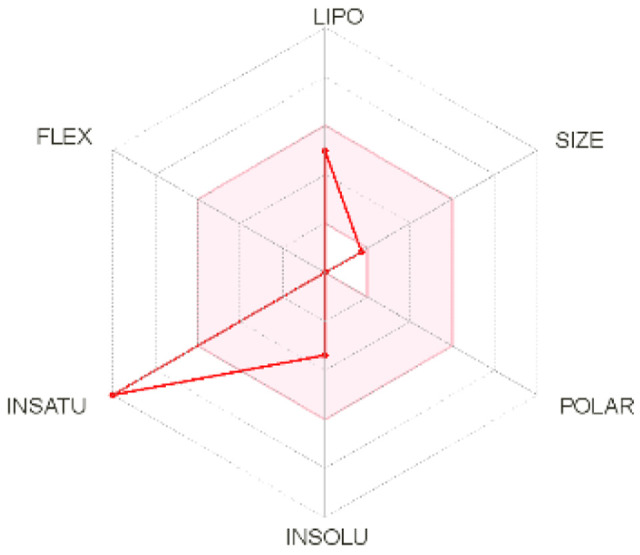	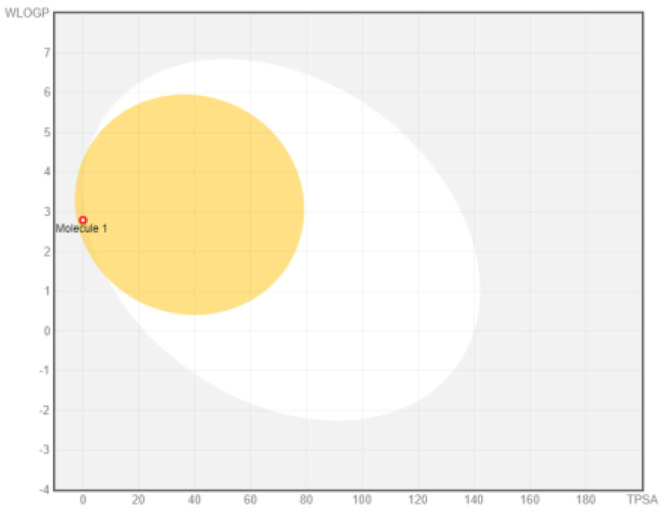
Flavonoid	Low	No	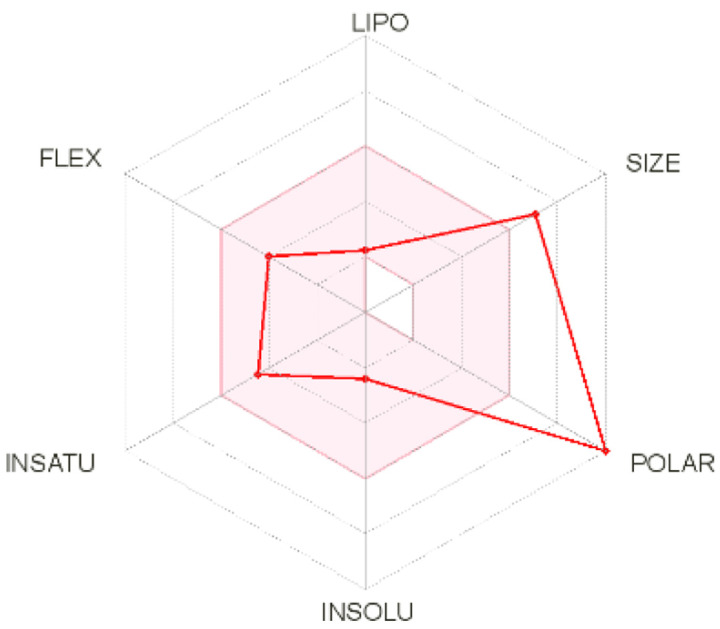	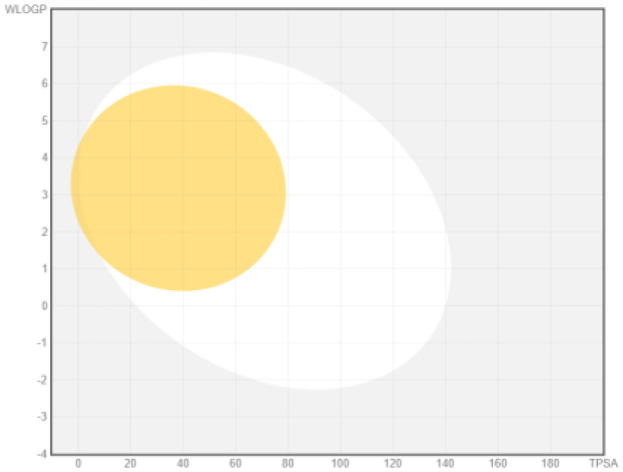
Phycocyanin	High	No	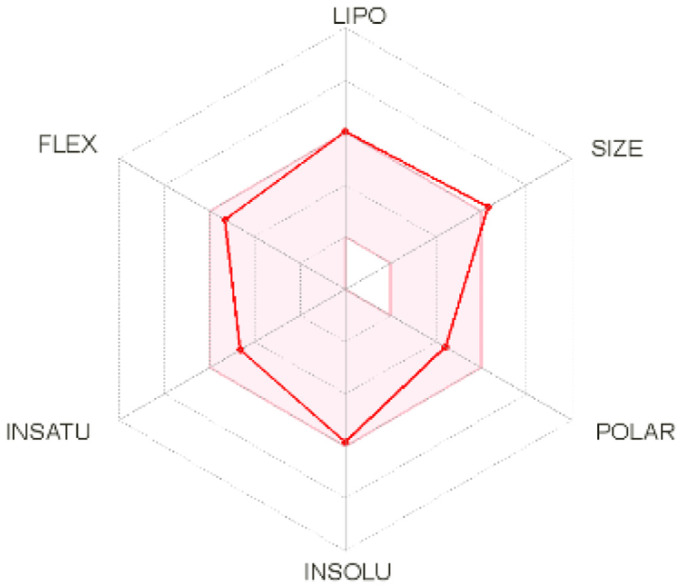	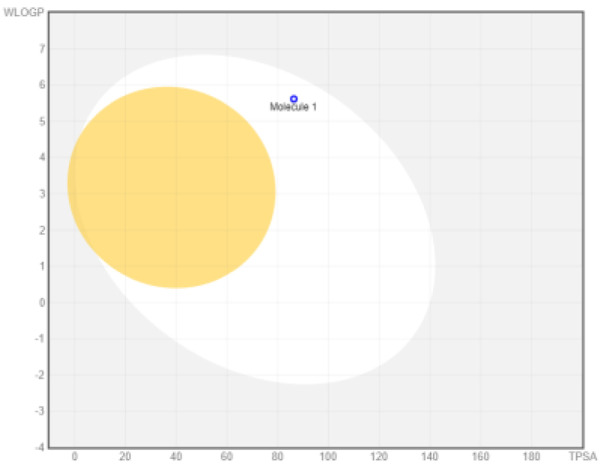

Note: GI: gatrointestinal; BBB: blood-brain barrier; LIPO: lipophilicity; FLEX: flexibility; INSATU: unsaturation; INSOLU: insolubility; POLAR: polarity; SIZE: molecular weight.

Table 2 and Table 3 demonstrate that phenol, azulene, and phycocyanin comply with Lipinski or drug-likeness laws. Drug-likeness is a term used to explain how *in vivo* molecular properties are influenced by compounds’ physicochemical properties. This research indicates that the substance will spread well to all parts of the body to play an active role as a drug ^79^. The physicochemical properties obtained from molecular structures are used by most drug-likeness testing laws and compare such properties with the medicines that have been reported. The Lipinski rule is one of the most used rules ^80^. The rule of five was developed to set drugability guidelines for new molecular entities (NMEs) ^81^. Therefore, the rule suggests that molecules, whose properties fall outside of these boundaries, are unlikely to become orally bioavailable drugs ^82^. As drug candidates, phenol, azulene, and phycocyanin have excellent potential. This calculation is based on a molecular weight (MW) value of less than 500 g mol ^-1^, an acceptor of hydrogen bonds of less than 10, a donor of hydrogen bonds of less than five, a surface area of topology (TPSA) of less than 140 Å, and a LogP of less than five.

**Table 2.** Physicochemical parameters of the analysis compound predicted by Swiss ADME.

Compound	MW (g.mol ^-1^)	HA	AHA	RB	HBA	HBD	MR	TPSA	L
Phenol	94.11	7	6	0	1	1	28.46	20.23	1.24
Azulene	128.17	10	10	0	0	0	43.06	0.00	2.07
Flavonoid	594.52	42	16	6	15	10	138.73	260.20	2.23
Phycocyanin	526.71	39	5	8	3	3	174.47	86.35	4.24

Note: MW: molecular weight; HA: heavy atoms; AHA: aromatic heavy atoms; RB: rotatable bonds; HBA: hydrogen bond acceptor; HBD: hydrogen bound donor; MR: molar refractivity; TPSA: topology polar surface area (Å²); L: lipophilicity

**Table 3.** Druglikeness property using Lipinski Rule of Five.

Compound	Molecular mass less than 500 Dalton	High lipophilicity (expressed as LogP less than 5)	Less than 5 hydrogen bond donors	Less than 10 hydrogen bond acceptors	Molar refractivity should be between 40–130	Conclusion
Phenol	Yes	Yes	Yes	Yes	No	Yes
Azulene	Yes	Yes	Yes	Yes	Yes	Yes
Flavonoid	No	Yes	No	No	No	No
Phycocyanin	No	No	Yes	Yes	No	Yes

## Conclusion

*Ocimum basilicum* intervention significantly increased total phenol, phycocyanin and antioxidant activity in *S. platensis*. However, total flavonoid content did not differ significantly in untreated *S. platensis* controls compared to treated. Bioactive compounds after microencapsulation showed the lowest values. Microencapsulation of phycocyanin with maltodextrin and gelatin showed high encapsulation efficiency values. Hence, *S. platensis* treated freeze-dried *O. basilicum* has potential as a functional foods and pharmaceutical product.

## Data availability

### Underlying data

Figshare: Underlying data for ‘ *Ocimum basilicum* (kemangi) intervention on powder and microencapsulated *Spirulina platensis* and its bioactive molecules’, https://doi.org/10.6084/m9.figshare.14291069.v3 ^40^

This project contains the following underlying data:

- Data file 1. Flavonoid content from the intervention of *O. basilicum* on *S. platensis* with microencapsulation.
- Data file 2. Antioxidant activity from the intervention of *O. basilicum* on *S. platensis* with microencapsulation.
- Data file 3. Encapsulation efficiency of phenol, flavonoid, antioxidant and phycocyanin content from the intervention of *O. basilicum* on *S. platensis* with microencapsulation.
- Data file 4. Phycocyanin content from the intervention of *O. bacilicum* on *S. platensis* with microencapsulation.
- Data file 5. Statistical analysis by SPSS v.22 on bioactive compounds.
- Data file 6. Total phenol content from the intervention of *O. basilicum* on *S. platensis* with microencapsulation.

Data are available under the terms of the Creative Commons Attribution 4.0 International license (CC BY 4.0).
