# Supplementary material for: Ocimum basilicum (kemangi) intervention on powder and microencapsulated  Spirulina platensis and its bioactive molecules
Source: F1000Res. 2022 Mar 14;10:485. Originally published 2021 Jun 18. [Version 3] doi: 10.12688/f1000research.52394.3 (PMC8758973; doi:10.12688/f1000research.52394.3)
Supplement: Peer review history

# Peer review: Reviewer response for version 3

**DOI:** 10.5256/f1000research.121649.r127388

**Published:** 2022-03-14

**Authors:** Made Airanthi K. Widjaja-Adhi

**Affiliations:** 1 Department of Pharmacology, School of Medicine, Case Western Reserve University, Cleveland, OH, USA

The authors have addressed all concerns. Therefore, this reviewer agrees the published revision in F1000Research is indexable.

Is the work clearly and accurately presented and does it cite the current literature?

Partly

If applicable, is the statistical analysis and its interpretation appropriate?

Yes

Are all the source data underlying the results available to ensure full reproducibility?

Yes

Is the study design appropriate and is the work technically sound?

Partly

Are the conclusions drawn adequately supported by the results?

Yes

Are sufficient details of methods and analysis provided to allow replication by others?

Partly

Reviewer Expertise:

Molecular biology, bioactive compounds, lipid metabolism, carotenoids/retinoids homeostasis, pharmacology.

I confirm that I have read this submission and believe that I have an appropriate level of expertise to confirm that it is of an acceptable scientific standard.

# Peer review: Reviewer response for version 2

**DOI:** 10.5256/f1000research.78756.r99214

**Published:** 2022-01-13

**Authors:** Made Airanthi K. Widjaja-Adhi

**Affiliations:** 1 Department of Pharmacology, School of Medicine, Case Western Reserve University, Cleveland, OH, USA

The authors have addressed the points raised by the reviewers sufficiently and improved the quality and transparency of the manuscript. I recommend the revised manuscript for indexing after minor revisions as listed below:

1.  Page 4 of 18: Determination of encapsulation efficiency.  Please change the *"in"* to *"is"* and add *"the"* before microcapsule in this sentence:
  "Where total coated active compounds **in** the total active compounds such as phycocyanin, phenol, flavonoid and antioxidant in microcapsule (MSB sample)".
2. Figures numbering should be in the sequence as it first appears in the text. Please change accordingly.
3. Page 5 of 18: Please add figure numbers after the statements:
  “The DSB sample can increase the total phenol 49.50% and antioxidant activity 12.67% of *S. platensis* **(Figure ?)**”.
  “However, total flavonoid is not significantly different with *O. basilicum* intervention on *S. platensis* **(Figure ?)**”.
4. Page 5 of 18: The statement, “The MSB sample is effective in phycocyanin protection and antioxidant activity that seen an encapsulation efficiency value (Figure 5). The results of the total phenol, flavonoid, and antioxidant activity of *O. basilicum* extract for 117.24 ± 8.06 mg GAE/g, 7.04 ± 0.18 mg QE/g and 94.93 ± 2.24%, respectively” is confusing. Please re-phrase.
5. Page 5 of 18: Please delete the first “is” in this sentence:
  “This **is** encapsulation is less effective in microencapsulation of polyphenol compounds such as phenol and flavonoid (Figure 5)”

Is the work clearly and accurately presented and does it cite the current literature?

Partly

If applicable, is the statistical analysis and its interpretation appropriate?

Yes

Are all the source data underlying the results available to ensure full reproducibility?

Yes

Is the study design appropriate and is the work technically sound?

Partly

Are the conclusions drawn adequately supported by the results?

Yes

Are sufficient details of methods and analysis provided to allow replication by others?

Partly

Reviewer Expertise:

Molecular biology, bioactive compounds, lipid metabolism, carotenoids/retinoids homeostasis, pharmacology.

I confirm that I have read this submission and believe that I have an appropriate level of expertise to confirm that it is of an acceptable scientific standard.

# Response

**Published:** 2022-02-23

**Authors:** Yuliani Yuliani

**Affiliations:** Diponegoro University, Indonesia

We would like to thank the reviewer for the time and willingness to assess the quality of our manuscript. In the new version our manuscript, suggested changes in the all section has been added according to the reviewer’s recommendation.

# Peer review: Reviewer response for version 2

**DOI:** 10.5256/f1000research.78756.r99213

**Published:** 2021-11-09

**Authors:** Joko Santoso

**Affiliations:** 1 Department of Aquatic Products Technology, Faculty of Fisheries and Marine Sciences, IPB University, Bogor, Indonesia

The author already improved the article base on the suggestions from the reviewer (me). Thank you very much for your kind cooperation.

Is the work clearly and accurately presented and does it cite the current literature?

Yes

If applicable, is the statistical analysis and its interpretation appropriate?

Partly

Are all the source data underlying the results available to ensure full reproducibility?

Yes

Is the study design appropriate and is the work technically sound?

Partly

Are the conclusions drawn adequately supported by the results?

Yes

Are sufficient details of methods and analysis provided to allow replication by others?

Yes

Reviewer Expertise:

Processing of aquatic organisms

I confirm that I have read this submission and believe that I have an appropriate level of expertise to confirm that it is of an acceptable scientific standard.

# Peer review: Reviewer response for version 1

**DOI:** 10.5256/f1000research.55668.r87948

**Published:** 2021-07-21

**Authors:** Made Airanthi K. Widjaja-Adhi

**Affiliations:** 1 Department of Pharmacology, School of Medicine, Case Western Reserve University, Cleveland, OH, USA

The manuscript by Yuliani *et al.* presents the effect of the intervention of *Ocimum basilicum* on freeze-dried and microencapsulated *Spirulina platensis*. In general, I find this manuscript provides an important contribution in developing palatable and higher bioactive compounds in *S. platensis*. However, I have outlined below a few issues that need to be addressed before publication, and some more minor comments and suggestions to further improve the quality of the manuscript.

**Major:**

1. The methods section is not clear. It is not clear whether *O. basilicum* alone was freeze-dried and/or microencapsulated, or the *S. platensis* alone, or is it after the intervention of *O. basilicum* to *S. platensis?* Example: In the sub-methods title “Intervention of *Ocimum basilicum* on *S. platensis*”, the authors stated “…freeze-dried sample (DSB) and microencapsulation sample (MSB) of *S. platensis*…”, while in the sub-methods title “Preparation of freeze-dried *Ocimum basilicum*”, the authors described that both *O. basilicum *and *S. platensis* were freeze-dried using a freeze dryer, and followed by a description of the “Microencapsulation of freeze-dried *Ocimum basilicum*”. Please clarify and make changes to the methods section as appropriate.
2. In the abstract and introduction, the authors stated that one of the aims of the studies is to reduce unpleasant odours of *S. platensis* by intervention with *O. basilicum*. However, the organoleptic properties were not presented and discussed.
3. The authors tried to predict the ADME of the bioactive compounds in the product ( *Ocimum basilicum - Spirulina platensis*) by using the SwissADME. However, as this approach can only predict pharmacokinetics, drug-likeness of small molecules, the authors use four main compounds found in the product namely phenol, azulene, flavonoid (not clear on which flavonoids), and phycocyanin (the structure of each compound used for analysis need to be added to Table 7). While this information provides a prediction of an individual molecule of the complex bioactive compounds in *Ocimum basilicum – Spirulina platensis*, it does not provide any additional insight to the manuscript as matrix-derived combination influences ADME. *In vivo* study is needed to determine the bioavailability of *Ocimum basilicum* intervention on freeze-dried and microencapsulated *Spirulina platensis*. If the authors wish to include this data in the current study, additional discussion in limitations and future studies include the need for *in vivo *study are needed.
4. In the method section, it is not clear how the encapsulation efficiency was determined. Do the authors analyze the active compounds in both the free and encapsulated fractions? If yes, please describe the procedure and parameter of separation of the free active compound from the encapsulation.
5. In the discussion section, it is not clear how this research indicates a synergistic interaction between phycocyanin and total phenols in antioxidant activities (page 9 of 16). No data support this statement.
6. Figure 4 is data on encapsulation efficiency. In the discussion (Page 9 of 16), by using the information in Figure 4, the authors stated the effect of different cultivation methods of *S. platensis* on phycocyanin content. No data support this statement. 

**Minor:**

1. In the result section paragraph 2 (page 4 of 16), please add a description of the result of MSB.
2. In the method section, microencapsulation, please describe what is the total solid content of coating materials used in the study.
3. In the discussion section paragraph 3 (page 6 of 16), please be clear on what bioactive compounds are the authors referring to and for which treatment.
4. In the discussion section paragraph 4 (page 7 of 16), please describe “…bioactive colored …”
5. In the introduction and abstract, *O. basilicum* intervention to *S. platensis* is to increase consumption and increase bioactive compounds and their potential for a functional food product. The conclusion state as a potential for a pharmaceutical product. Please be consistent.  
6. In Figure 2, B = *O. basilicum* leaf extract was analyzed. Please describe B in the method section.

The paper would benefit from a more detailed discussion regarding the organoleptic properties of how the *Ocimum basilicum* intervention reduces unpleasant odours as outlined in the comments above.

 An additional suggestion that will improve the presentation of the manuscript is listed below:

1. The result section should focus on the main finding and not the statistical analysis. Figure 1 and Table 1-6 can be added to the supplemental section.
2. The authors need to check the accuracy of the statement in the result section’s first sentence (page 4 of 16). Figure 1 is a standard calibration curve use to calculate total phenol of samples as gallic acid equivalent (GAE) and total flavonoid of samples as quercetin equivalent (QE) but not for antioxidant activity. Additionally, this information is more suitable to be in the method section and not the result section. Please also refer to my additional suggestion point 1.
3. Citation in the discussion should always be referred to the result of the study, either it is in accordance or opposition with the current finding. In the discussion section page 7, the authors cite that microencapsulation can maintain the stability of flavonoids from processing effects that cause degradation. Please elaborate more on how this statement is in the context of the current finding.  

Is the work clearly and accurately presented and does it cite the current literature?

Partly

If applicable, is the statistical analysis and its interpretation appropriate?

Yes

Are all the source data underlying the results available to ensure full reproducibility?

Yes

Is the study design appropriate and is the work technically sound?

Partly

Are the conclusions drawn adequately supported by the results?

Yes

Are sufficient details of methods and analysis provided to allow replication by others?

Partly

Reviewer Expertise:

Bioactive compounds, lipid metabolism, carotenoids/retinoids homeostasis, pharmacology

I confirm that I have read this submission and believe that I have an appropriate level of expertise to confirm that it is of an acceptable scientific standard, however I have significant reservations, as outlined above.

# Response

**Published:** 2021-10-26

**Authors:** Yuliani Yuliani

**Affiliations:** Diponegoro University, Indonesia

We would like to thank the reviewer for the time and willingness to assess the quality of our manuscript. In the new version our manuscript, suggested changes in the all section has been added according to the reviewer’s recommendation, except for the SwissADME analysis which will be carried out in next studies.

 This research, SwissADME is used to predict chemical structure homology. SwissADME is used as the initial stage for further research with experimental animals. The proof will be carried out in future research and publications on in vivo.

# Peer review: Reviewer response for version 1

**DOI:** 10.5256/f1000research.55668.r87945

**Published:** 2021-07-12

**Authors:** Joko Santoso

**Affiliations:** 1 Department of Aquatic Products Technology, Faculty of Fisheries and Marine Sciences, IPB University, Bogor, Indonesia

This work presents to enriche *Sprirulina platensis* with *Ocium basilicum* by soaking method, and evaluated their characteristic after being freeze-dried and Microencapsulation of freeze-dried.

 The manuscript is recommended to be revised as follows:

- Authors stated the intervention of *O. basilicum *on *S. platensis *(DSB) significantly increases total phenol by 48.7% (in Page 1); Page 4 (Results) states The DSB sample can increase the total phenol 49.50% (I calculated it, that 49.5% is true value); antioxidant activity 12.66% (page 4 results), I calculated 12.67% (Please check it).
- Fig 1 shows the calibration, this is the method of how to get the value of total phenol and flavonoid content. Fig 1 should be deleted.
- The results, Tukey HSD (Table 2 2-6) (Page 4, 5, 6, 7) statistic analysis, it is not necessary to written in the manuscript, please delete.
- Fig 2. In the analysis data process (statistic analysis), B ( *Ocium basilicum*) does not include the factors studied in accordance with the research objectives, B is excluded from the data analysis process (statistics) (See Fig 3, the ANOVA like this). The value of phenol, flavonoid and antioxidant activity of B can be written in the paragraph). Please re-analyse the statistics (ANOVA).
- (5) Fig 2, please separate into three figures and complete the description of the y-axis (the unit of y-axis such as %, mgQE/g).

**Minor revision:**

- *"Spirulina platensis *is a blue-green microalgae" (Page 2) - it should be microalga (single).
- "A freeze-dried sample (DSB) and microencapsulation sample (MSB) of *S. platensis *were soaked (page 4)" - add time for soaking (how long).
- "Gallic acid was used as standard and was read at λ=739 nm using a spectrophotometer" - add the type of spectrophotometer.
- "40 mg of sample was added" - sample of 40 g.
- Make all figures clear with same font, x-axis and y-axis description should be added in the each figure.
- "Algae are a valuable source of proteins and phenol compounds" - A algae means macroalgae, be careful with this statement.

Is the work clearly and accurately presented and does it cite the current literature?

Yes

If applicable, is the statistical analysis and its interpretation appropriate?

Partly

Are all the source data underlying the results available to ensure full reproducibility?

Yes

Is the study design appropriate and is the work technically sound?

Partly

Are the conclusions drawn adequately supported by the results?

Yes

Are sufficient details of methods and analysis provided to allow replication by others?

Yes

Reviewer Expertise:

Processing of aquatic organisme

I confirm that I have read this submission and believe that I have an appropriate level of expertise to confirm that it is of an acceptable scientific standard, however I have significant reservations, as outlined above.

# Response

**Published:** 2021-10-26

**Authors:** Yuliani Yuliani

**Affiliations:** Diponegoro University, Indonesia

We would like to thank the reviewer for the time and willingness to assess the quality of our manuscript. In the new version our manuscript, suggested changes in the all section has been added according to the reviewer’s recommendation.
